# A Case of 'Neptune’s Fix Elixir': The Dangerous Consequences of Unregulated Use of Tianeptine in Over-the-Counter Products

**DOI:** 10.7759/cureus.55120

**Published:** 2024-02-28

**Authors:** Eduardo D Espiridion, Maya Qutob, Priscilla Lozano

**Affiliations:** 1 Psychiatry, Drexel University College of Medicine, Philadelphia, USA; 2 Psychiatry, Reading Hospital, West Reading, USA; 3 Psychiatry, Tower Health Medical Group, Reading , USA; 4 Psychiatry, Drexel University College of Medicine, Reading, USA

**Keywords:** substance abuse, over the counter, illicit drugs, neptune's fix elixir, tianeptine

## Abstract

Tianeptine is an atypical tricyclic antidepressant approved for the treatment of major depressive disorder in some European, Asian, and Latin countries. Along with its serotonergic properties, tianeptine also acts as a full agonist at the mu-opioid receptor, creating sensations of euphoric highs and significant risks of addiction and withdrawal. For this reason, along with increased reports of adverse effects and fatalities, tianeptine has not been approved in the US. Despite this, tianeptine continues to be accessible through unregulated online stores and small retailers under street names such as Zaza, Tia, Tianna, 'gas-station dope', and a product not mentioned in the literature previously: Neptune’s Fix Elixir. In this report, we discuss the case of a 34-year-old male who presented to the ED via EMS after being found unresponsive secondary to the ingestion of Neptune’s Fix Elixir, whose main active ingredient is tianeptine.

## Introduction

Tianeptine, an atypical tricyclic antidepressant, is an agent used in the treatment of major depressive disorder, anxiety, and other medical conditions, including asthma and irritable bowel syndrome [1-4]. It is approved for use in some European, Asian, and Latin American countries; however, it is not approved for use by the FDA in the US. Previous studies have classified tianeptine as a serotonin reuptake enhancer [5]. Recent studies suggest that the drug has additional mechanisms of action, including full agonism at the mu-type opioid receptor (MOR) [6]. Furthermore, tianeptine has also been found to have action on dopamine (D2/D3) and glutamate (NMDA, AMPA) receptors [7,8]. The glutamate modulation is thought to be responsible for its antidepressant effects, but its MOR activation has been repeatedly implicated in its abuse potential and dependency [6].

The availability of dangerous, psychotropic substances at gas stations and e-commerce stores has increasingly been of national concern [9-11], and unfortunately, the public’s accessibility to tianeptine is no exception. Although tianeptine is not approved for use in the US by the FDA, it can still be found in gas stations, e-commerce stores, and convenience stores marketed as a supplement or 'cognitive enhancer'. It is widely available for purchase, even to individuals under the age of 18, under the names Pegasus, ZaZa Red, Tianna Red, and, as will be reported for the first time in this case study, Neptune’s Fix Elixir. This drug is known by the nickname 'gas station heroin' due to its agonist activity on MOR and its common misuse.This report discusses the case of a 34-year-old male who presented to the ED via EMS after being found unresponsive secondary to the ingestion of Neptune’s Fix Elixir, which contains tianetptine as its main ingredient.

## Case presentation

A 34-year-old male with a past psychiatric history of polysubstance abuse (heroin, kratom, marijuana, and tobacco) and a reported history of unspecified depression presented at a local community hospital ED via EMS after being found unresponsive in the passenger's seat of his partner's vehicle. Along with the patient, empty bottles of Neptune’s Fix Elixir were found nearby. While in the field, the patient was administered naloxone with no response. The patient was promptly intubated in the field due to concerns about difficulty maintaining their airway and was transferred to the ED for further care. On presentation to the ED, he was found to have a Glasgow coma score (GCS) of 3T and tachycardia, with fixed and dilated pupils. Table 1 shows laboratory results that revealed leukocytosis, elevated creatinine, hyperglycemia, and severe metabolic acidosis with an elevated anion gap in the absence of an osmolar gap. The urine toxicology screen was significant for tetrahydrocannabinol, but ethanol, salicylate, and acetaminophen levels were normal.

**Table 1:** Relevant laboratory findings on admission

Laboratory test	Results	Reference values
Serum sodium	139 mmol/L	136 - 145 mmol/L
Serum potassium	3.9 mmol/L	3.5 - 5.1 mmol/L
Serum calcium	8.7 mg/dL	8.7 - 10.4 mg/dL
Serum creatinine	1.12 mg/dL	0.73 - 1.18 mg/dL
Blood urea nitrogen (BUN)	12 mg/dL	9 - 23 mg/dL
Phosphorus	4.8 mg/dL	2.4 - 5.1 mg/dL
Anion gap	7 mmol/L	4-12 mmol/L
Glucose	89 mg/dL	74 - 99 mg/dL
Hemoglobin	14.1 g/dL	14.0 - 17.5 g/dL
Hematocrit	43.3%	39.0% - 53.0%
White blood cell count	32.7 x 10^9^/L	4.8 - 10.8 x 10^9^/L
Mean corpuscular volume (MCV)	95.2 fl	80-100 fl
Fentanyl screen, urine	Negative	Negative
Benzodiazepine screen, urine	Negative	Negative
Marijuana screen, urine	Positive	Negative

Beta-hydroxybutyrate was checked in the setting of hyperglycemia but was found to be within normal limits. Neuroimaging was unremarkable for acute intracranial pathology, and the chest X-ray was unrevealing of acute abnormalities. The patient was admitted for management of acute hypoxic respiratory failure in the setting of a suspected overdose. Per the recommendations of poison control, the patient was treated with aggressive supportive care, including naloxone as indicated and a trend EKG to monitor QTc intervals with the administration of magnesium and bicarbonate when necessary.

**Figure 1:**
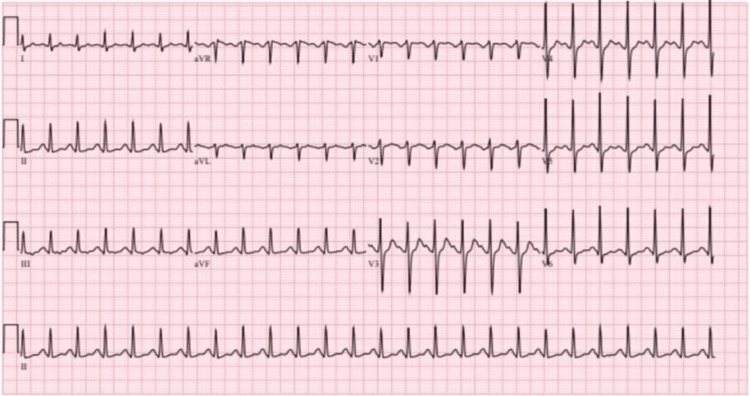
The EKG on admission shows a QTc of 524 milliseconds

The EKG reported a corrected QT interval (QTc) of 524 milliseconds on admission (Figure 1), which trended downward throughout the patient’s hospitalization. Collateral information revealed that the patient had not been adherent to his psychotropic medications for months in the context of extraneous psychosocial stressors, including interpersonal conflict with family members. The patient was extubated on the following day of admission, was in stable condition, and underwent further psychiatric evaluation, where he reported drinking Neptune’s Fix Elixir with the intent of receiving similar euphoric effects to stimulants but had never used it before. He reported a history of three prior psychiatric hospitalizations during childhood, one being a suicide attempt by drug overdose, but none recently. Previous substance abuse included kratom, marijuana, and methamphetamine. It remains unclear the last time any of these substances were used. Family history was significant for depression, suicide, and addiction disorders. The patient denied that this incident was a suicide attempt. No further information could be obtained as the patient left the hospital against medical advice.

## Discussion

In the US, tianeptine's abuse potential and its lack of regulation in over-the-counter products as the active ingredient pose a large safety concern for the public [12]. Considering tianeptine’s pharmacologic profile as a glutamate modulator, i.e., both a full MOR and a weak delta opioid receptor agonist, its pleasurable euphoria, accessibility, and lack of regulation leave individuals vulnerable to unsuspecting abuse, dependence, and overdose. In particular, those with a history of substance abuse are the largest population at risk [13]. In this study, we identified an over-the-counter product called Neptune’s Fix Elixir, the main ingredient of which is tianeptine, widely sold through online commerce and convenience stores, including gas stations. Tianeptine withdrawal is known to have a similar withdrawal profile to opioid withdrawal [14]. The most commonly reported adverse effects of tianeptine include agitation, nausea, vomiting, tachycardia, hypertension, diarrhea, tremor, and diaphoresis. Severe adverse health effects, including respiratory depression, severe sedation, and death, have occurred from the misuse of tianeptine [15].

From 2020 to 2022, more than 600 calls were made to poison control centers after exposure, and five deaths occurred as a result [15]. The Centers for Disease Control and Prevention report a statistically significant increase in tianeptine exposure calls related to intentional abuse and misuse between 2014 and 2017, with the majority of these calls (91.2%) coming from healthcare providers [15]. As of September 2021, Florida has joined eight other states, including Alabama, Georgia, Indiana, Kentucky, Michigan, Mississippi, Ohio, Oklahoma, and Tennessee, to classify tianeptine as a Schedule 1 controlled substance [15]. Despite state-wide attempts to control the use of tianeptine, it continues to be used in products such as Neptune’s Fix Elixir nationwide. Advertised as a legal herbal product, one website describes Neptune's Fix Elixir as “the ultimate solution to elevating your mood and conquering life’s challenges." It is available for purchase at different "strengths," with no mention of the dangers of its main ingredient. The website goes as far as to guarantee the customer that their “rigorous manufacturing process guarantees that every drop (of Neptune’s Fix Elixir) is free from harmful additives and impurities." In November 2023, the FDA issued a warning against this very product, stating that “FDA has received severe adverse event reports after use of Neptune’s Fix products, including seizures and loss of consciousness” [16].

Tolerance and withdrawal have been reported, as well as neonatal abstinence syndrome after tianeptine dependence during pregnancy [17]. Although the misuse of this product has been on the rise, there has not been much awareness of this product, including its active ingredient, within the medical community [18]. Although the abuse of tianeptine-containing products is on the rise, their existence remains elusive to the medical community within the United States. Therefore, healthcare professionals must be made aware of products with tianeptine and their accessibility and urged to screen vulnerable individuals for the possible use of these products. Furthermore, better regulation of this product should be implemented at the federal level to limit unregulated use. Lastly, directions regarding treating tianeptine toxicity must be investigated and outlined to guide management.

## Conclusions

In this case report, we highlight the case of a patient who overdosed on a tianeptine-containing gas station product known as Neptune’s Fix Elixir, a product that has yet to be reported in medical literature but is easily becoming a nationwide concern across the US due to its wide availability and propensity for misuse. Its abuse potential, potential lethal side effects, accessibility, and lack of regulation pose major public safety concerns. Medical professionals nationwide must be made aware of these products and other similar products to accurately educate patients on their dangers as well as identify cases of overdose related to them.
